# Supplementary material for: Spatio-Temporal Distribution of Aedes Albopictus and Culex Pipiens along an Urban-Natural Gradient in the Ventotene Island, Italy
Source: Int J Environ Res Public Health. 2020 Nov 10;17(22):8300. doi: 10.3390/ijerph17228300 (PMC7696970; doi:10.3390/ijerph17228300)
Supplement: Supplementary file 1 [file ijerph-17-08300-s001.zip › TableS1.pdf]

Table S1. Natural to urban transect represented by the percentage of buildings in a 250-meter buffer around traps.

IDsite	% Buildings in 250m buffer
1	0.79
2	1.14
4	1.63
6	2.47
3	3.47
9	3.84
5	4
7	5.54
8	13.38
10	20.34
