# Supplementary material for: Spatio-Temporal Distribution of Aedes Albopictus and Culex Pipiens along an Urban-Natural Gradient in the Ventotene Island, Italy
Source: Int J Environ Res Public Health. 2020 Nov 10;17(22):8300. doi: 10.3390/ijerph17228300 (PMC7696970; doi:10.3390/ijerph17228300)
Supplement: Supplementary file 1 [file ijerph-17-08300-s001.zip › TableS2.pdf]

Table S2. Pvalues of model parameters when different buffer sizes are considered when computing the percentage of buildings around traps.

Radius	intercept	Species	buildings	interaction
50m	0.00294	<0,0001	0.89356	0.77344
100m	0.00121	<0,0001	0.78974	0.90942
150m	0.00082	<0,0001	0.51858	0.85484
200m	<0,0001	<0,0001	0.00963	0.09017
250m	<0,0001	<0,0001	0.00023	0.00824
300m	<0,0001	<0,0001	0.00013	0.00275
350m	<0,0001	<0,0001	<0,0001	0.00064
400m	<0,0001	<0,0001	<0,0001	0.00029
450m	<0,0001	<0,0001	<0,0001	<0,0001
500m	<0,0001	<0,0001	<0,0001	<0,0001
