# Supplementary figures and images for: Spatio-Temporal Distribution of Aedes Albopictus and Culex Pipiens along an Urban-Natural Gradient in the Ventotene Island, Italy
Source: Int J Environ Res Public Health. 2020 Nov 10;17(22):8300. doi: 10.3390/ijerph17228300 (PMC7696970; doi:10.3390/ijerph17228300)

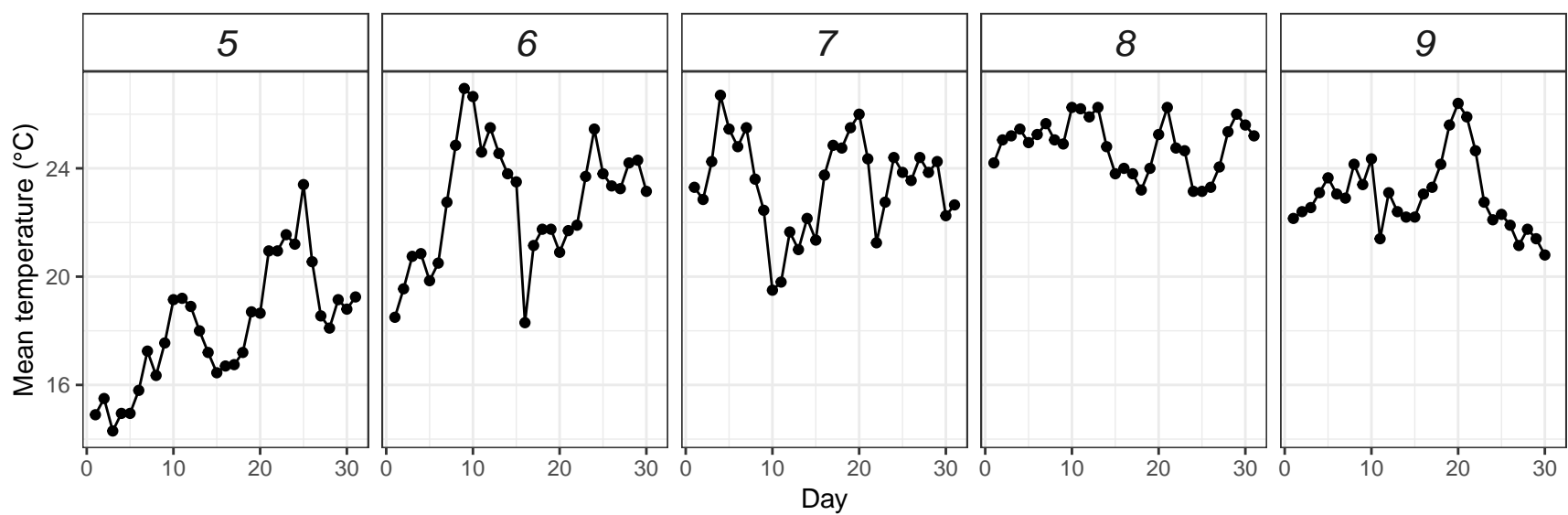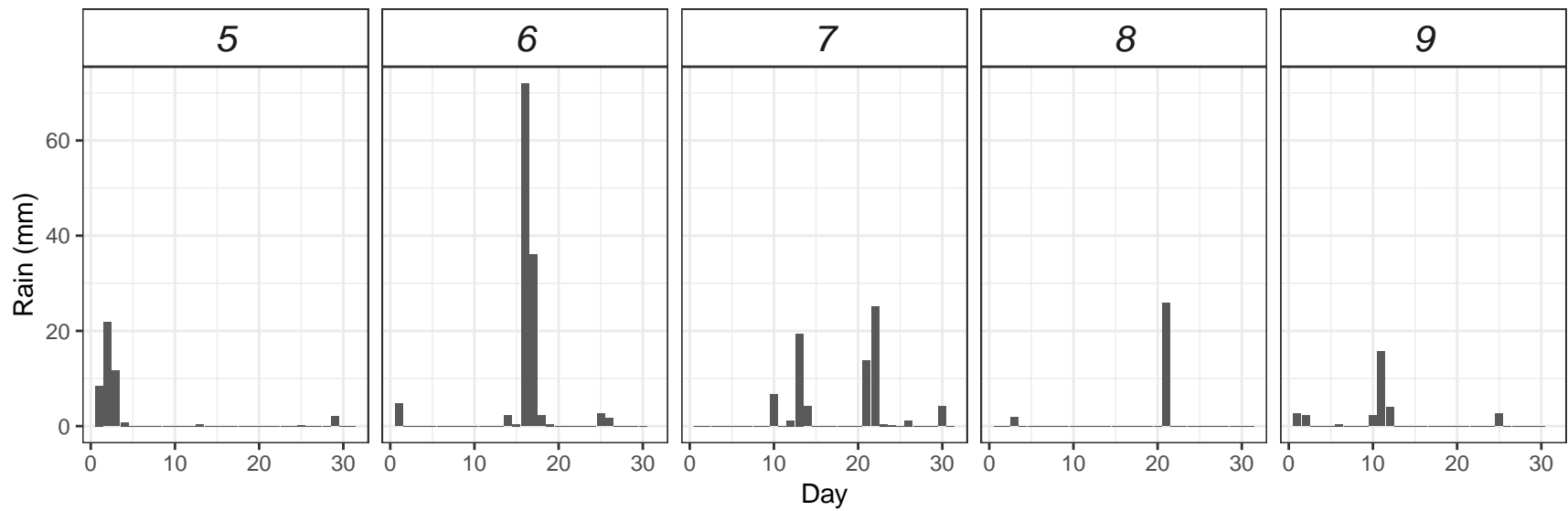

Supplement: Supplementary file 1 [file ijerph-17-08300-s001.zip › figS2.pdf]

Mean mosquito abundance

|     |     |     |     |      |
|-----|-----|-----|-----|------|
| 0.0 | 2.5 | 5.0 | 7.5 | 10.0 |
|-----|-----|-----|-----|------|

*Aedes*

*Culex*

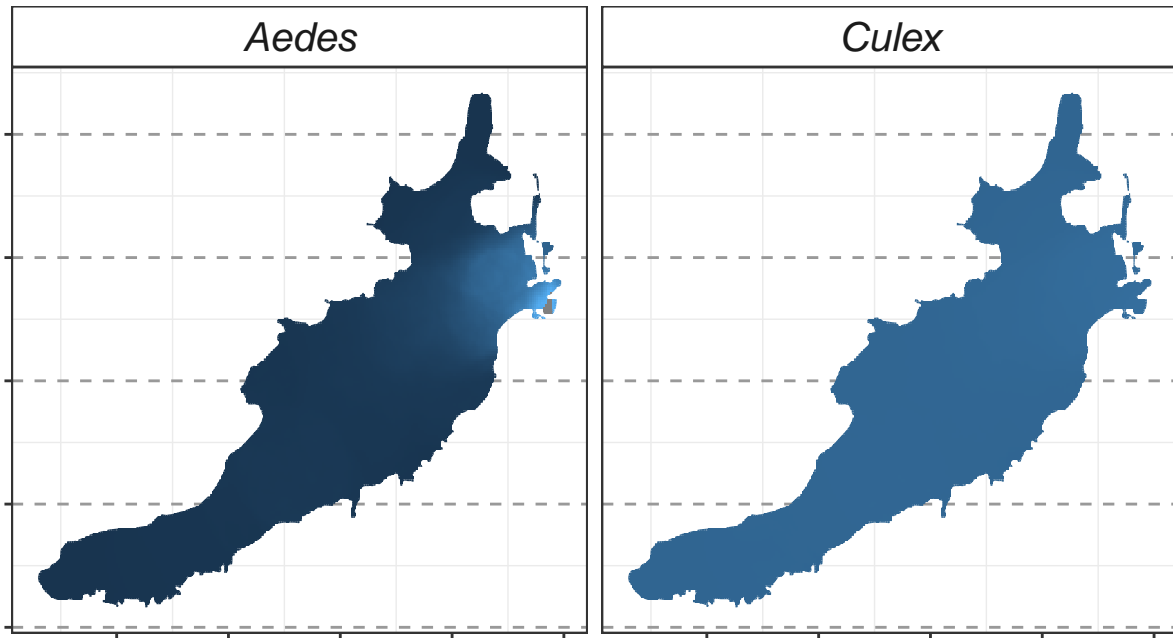

Supplement: Supplementary file 1 [file ijerph-17-08300-s001.zip › figS1.pdf]
